# Safety and efficacy of *N*-acetylcysteine (NAC) as an adjunct to standard treatment in patients with acute ischemic stroke: a randomized controlled pilot trial (NACTLYS)

**DOI:** 10.1038/s41598-023-49054-9

**Published:** 2024-01-11

**Authors:** Snigdha Komakula, Rohit Bhatia, Akhil Sahib, Ashish Upadhyay, Leve Joseph S, Ajay Garg, Vishnu V Y, Awadh Kishor Pandit, Deepti Vibha, Mamta Bhushan Singh, Manjari Tripathi, M. V. Padma Srivastava

**Affiliations:** 1 Department of Neurology, All India Institute of Medical Sciences (AIIMS), Ansari Nagar, New Delhi, 110029 India; 2 Department of Neuroradiology and Neurointervention, All India Institute of Medical Sciences, New Delhi, 110029 India; 3 Department of Biostatistics, All India Institute of Medical Sciences, New Delhi, 110029 India

**Keywords:** Neuroscience, Neurology

## Abstract

There is a pressing clinical need for thrombolytic agents that can effectively disaggregate arterial thrombi in acute ischemic stroke without significantly increasing the risk of bleeding. This pilot study aimed to investigate the safety and efficacy of *N*-acetylcysteine (NAC) as an adjunctive therapy to intravenous recombinant tissue plasminogen activator (rtPA or alteplase). A randomized, open-label, blinded assessor pilot study was conducted. Patients presenting with an acute ischemic stroke within 4.5 h from onset were randomized into two groups: intravenous NAC and rtPA or rtPA alone. Primary outcomes included intracerebral hemorrhage, symptomatic intracerebral hemorrhage, extracranial bleeding, and adverse reactions. Secondary outcomes comprised major neurological improvement assessed by (National Institute of Health Stroke Scale) NIHSS at 24 h, recanalization on first run of angiography in patients who underwent thrombectomy or on repeat vascular imaging at 24 h, modified Rankin scale, and three-month mortality. Forty patients were enrolled, with 21 receiving only rtPA and 19 receiving NAC with rtPA. Baseline characteristics were comparable among groups. No significant differences were observed in adverse events (*p* = 0.99), intracranial hemorrhage (*p* = 0.21), symptomatic intracerebral hemorrhage (*p* = 0.47), or extracranial bleeding (*p* = 0.21). Median NIHSS at 24 h was significantly lower in the intervention group (*p* = 0.03). Functional outcomes and three-month mortality were similar between groups (*p* = 0.85 and *p* = 0.99 respectively). The co-administration of *N*-acetylcysteine with alteplase did not significantly alter safety profiles, morbidity, or mortality at 3 months. While no substantial differences were noted, a slightly improved early neurological outcome was observed in the intervention arm. The study's findings were constrained by a small sample size, emphasizing the necessity for future large-scale trials to comprehensively evaluate the safety and efficacy of *N*-acetylcysteine as a thrombolytic agent in acute ischemic stroke.

Trial Registration Clinical Trials Registry India—CTRI/2019/05/019305.

## Introduction

Stroke is one of the leading causes of death and disability across the world. In acute ischemic stroke, although some brain tissue dies almost immediately, a significant amount of compromised brain tissue is potentially salvageable if blood supply can be restored^1^. Thrombolysis for acute ischemic stroke is a key intervention that can reduce disability. rtPA (recombinant tissue plasminogen activator) alone can achieve recanalization in approximately only 30%^2^ and recanalization rates can be as low as 6% in strokes with occlusion secondary to platelet-rich thrombi^3^.

During endovascular procedure, platelet-rich thrombi are more resistant to mechanical removal. High shear arterial thrombosis involves mainly von Willebrand Factor (VWF)-dependent platelet cross-linking^4^. Thus, proteolysis of VWF could be an efficient strategy to disaggregate arterial and platelet-rich thrombi. *N*-acetyl Cysteine (NAC) is acetyl derivative of the amino acid cysteine. It also has antioxidant and a free-radical scavenging activity that increases intracellular Glutathione (GSH), a major component of the pathways by which cells are protected from oxidative stress. The thrombolytic effect of NAC administration is mainly mediated by cleavage of the VWF that crosslinks platelets inside arterial thrombi^4^. *N*-Acetyl cysteine has been studied for safety and efficacy in animal models of ischemic and hemorrhagic stroke. Martinez and colleagues observed that NAC administration led to a rapid and significant reperfusion reaching up to 53% of the baseline cerebral blood flow (CBF) in mouse models with FeCl3-induced platelet-rich thrombi and thrombin induced formation of mixed thrombi in the middle cerebral artery (MCA), as measured by laser Doppler flowmetry^4^. A previous study on a rat stroke model where focal cerebral ischemia was produced by middle cerebral artery occlusion and pretreated with NAC at150 mg/kg, showed a 49.7% reduction in brain infarct volume and 50% reduction in the neurological evaluation score compared to untreated animals^5^. In an another middle cerebral artery occlusion mouse model, NAC dissolved in saline with 1% dimethyl sulfoxide (DMSO) was administered at 150 mg/kg body wt. intraperitoneally at 30 min before the onset of ischemia. Administration of NAC reduced the infarct volume (140.27 18.8 mm^3^), compared to the control group (226.0726.1 mm^3^; p¼0.0135)^6^. In the present study, we hypothesized that *N*-acetylcysteine (NAC) may reduce the size of Von Willebrand Factor (VWF) multimers that cross-link platelets inside arterial thrombi and promote thrombus dissolution leading to arterial recanalization^7^, especially in case of platelet-rich thrombi, based on in vitro study results and animal studies. The utilization of NAC, in addition to the currently employed thrombolytic agent, may be safe and could help achieve better recanalization, thereby promoting improved clinical recovery and reducing mortality.

## Methods

### Trial design

We conducted a prospective, randomized, open-label, blinded endpoint (PROBE), Phase 2 pilot study. This was conducted in the department of Neurology, All India Institute of Medical Sciences (AIIMS), New Delhi between 1st February 2019 and 30th April 2021. The study protocol was prospectively registered in clinical trials registry of India (CTRI) vide CTRI number CTRI/2019/05/019305 on 23/05/2019. All research was performed in accordance with relevant guidelines and regulations.

Patients with an acute ischemic stroke (AIS) presenting to the emergency services within 4.5 h of stroke onset with or without major vessel occlusion were included. Patients were screened for randomization into the study and were eligible if they could receive intravenous tissue plasminogen activator tPA (alteplase) within 4.5 h after the onset of ischemic stroke. The inclusion and exclusion criteria are summarized in Table 1.

**Table 1:** Inclusion and exclusion criteria.

Inclusion criteria	Exclusion criteria
1. Patients presenting with acute ischemic stroke within 4.5 h of stroke onset 2. Age ≥ 18 years and upto 80 years 3. Eligible to receive intravenous (IV) thrombolysis with tissue plasminogen activator tPA and where indicated 4. Endovascular thrombectomy can commence within 6 h of stroke onset and in highly selected cases up to 24 h based on imaging inclusion criteria	1. Any intracranial hemorrhage identified by CT or MRI 2. Pre-stroke mRS score of ≥ 2 (indicating previous disability) 3. Hypodensity in > 1/3 MCA territory on non-contrast CT 4. Presence of carotid dissection 5. Pregnant women 6. Previous stroke within last three months 7. Recent history or clinical presentation of ICH, subarachnoid hemorrhage (SAH), arterio-venous (AV) malformation, aneurysm or cerebral neoplasm (at the discretion of investigator) 8. Current use of oral anticoagulants and a prolonged prothrombin time (INR > 1.7) or use of heparin, except for low dose subcutaneous heparin, in the previous 48 h and a prolonged APTT or use of glycoprotein IIb-IIIa inhibitors within the past 72 h 9. Clinically significant hypoglycemia, uncontrolled hypertension defined by a blood pressure > 185 mmHg systolic or > 110 mmHg diastolic on at least 2 separate occasions at least 10 min apart 10. Hereditary or acquired hemorrhagic diathesis. gastrointestinal or urinary bleeding within the preceding 21 days 11. Major surgery within the preceding 14 days which posed risk 12. Any condition that in the judgment of the investigator could impose hazards to the patient if study therapy is initiated or affect the participation of the patient in the study

### Trial interventions and measures

In this pilot trial, it was planned to randomize 50 patients for participation in the study. Randomization was done using computer generated random sequence allocation in a 1:1 ratio to either receive intravenous rtPA and intravenous N Acetyl Cysteine or IV rtPA alone. Allocation was concealed in a sealed opaque envelope and the intervention was open label. All eligible patients in both arms received intravenous tissue plasminogen activator (rtPA; alteplase) at a standard dose of 0.9 mg per kilogram not exceeding 90 mg. *N*-acetylcysteine was given intravenously at a dose of 150 mg/kg. This dosage is approved for use in human cases of paracetamol poisoning and has also been examined in a limited number of preclinical studies, such as those conducted by Sekhon et al^5,8^, where *N*-acetylcysteine was explored as a thrombolytic agent. All those examiners involved in the subsequent clinical and imaging assessment of outcomes were blinded to the treatment allocation. All eligible patients with large vessel occlusion (LVO) received endovascular therapy. Baseline physical examination, National Institutes of Health Stroke Scale (NIHSS) and pre stroke mRS (modified Rankin scale) were assessed. Computed Tomography (CT)/CT Angiography Brain was performed to assess eligibility for intravenous rtPA as part of standard care. After satisfying the inclusion and exclusion criteria, patients who were eligible to receive rtPA (alteplase) were recruited in to the study.

Details of the baseline characteristics including age, sex, presence or absence of risk factors including smoking, alcohol abuse, diabetes, hypertension, atrial fibrillation (AF), presence or absence of comorbidities including chronic kidney disease (CKD), coronary artery disease (CAD), hypothyroidism, rheumatic heart disease (RHD), prior stroke, malignancy, or any other relevant conditions like use of antiplatelets or oral anticoagulants were collected. Baseline clinical characteristics details like Glasgow Coma Scale (GCS), NIHSS, blood pressure measurement, imaging characteristics like ASPECTS score (Alberta Stroke Program Early Computed Tomography Score), vascular territory involved, presence or absence of occlusion, site of occlusion and TOAST (Trial of Org 10172 in Acute Stroke Treatment^9^) classification were collected. We also analyzed time metrics including onset of stroke to door time, door to CT time, onset to tPA time, door to tPA time, onset to NAC time, door to NAC time. In patients who underwent endovascular treatment, presence or absence of recanalization at first run of angiogram, onset to groin puncture time, door to groin puncture time, groin puncture to recanalization, type of device used, whether recanalization achieved or not and modified Thrombolysis in Cerebral Infarction (mTICI)^10^ grade of recanalization were noted. NIHSS at 24 h and ASPECTS on repeat CT Brain at 24 h were assessed for all patients. ﻿Routine hematological and biochemical evaluation was done for all patients.

### Outcomes

Primary outcomes included safety measures like any adverse reactions of IV administration of *N*-Acetyl cysteine, any intracerebral hemorrhage or symptomatic intracerebral hemorrhage as defined by The Safe Implementation of Thrombolysis in Stroke-Monitoring Study (SITS-MOST) criteria^11^ and any extracranial bleeding. Repeat imaging was usually performed after 24 h or earlier as required. Clinical assessment was done for each patient during the infusion period for any rash, angioedema, headache, vomiting, seizures, extracranial bleeding, altered sensorium or worsening neurological features up to 24–72 h.

Secondary outcomes included major neurologic improvement at 24 h (defined as a reduction from baseline of eight or more points on the NIHSS or a final NIHSS of zero), recanalization on first run of angiography in patients who underwent thrombectomy or on repeat vascular imaging at 24 h, morbidity as assessed by mRS at 90 days and mortality at 90 days. In patients with occlusion on vascular imaging at baseline and who had not undergone thrombectomy, repeat vascular imaging using time of flight (TOF) magnetic resonance angiography (MRA) was performed to assess recanalization after 24 h. mRS at three months was assessed either telephonically or during the in person visit to the clinic. An mRS ≤ 2 was taken as a good outcome. These outcomes were assessed by a blinded assessor.

### Statistical analysis

The statistical analysis was conducted using Stata, version 14. Continuous variables conforming to a normal distribution were compared utilizing the independent t-test, while non-normally distributed continuous variables were assessed using the Wilcoxon rank-sum test. Categorical variables were analyzed employing the chi-square or Fisher exact test. Kaplan–Meier analysis was performed to evaluate survival patterns, with the log-rank test applied to discern differences in survival patterns between the two groups. Statistical significance was defined as a two-tailed p-value less than 0.05.

### Ethical approval

The study protocol was approved by the “Institute Ethics committee For Post Graduate Research” of All India Institute of Medical Sciences (AIIMS), New Delhi (vide letter number IECPG-74/23.01.2019).

### Informed consent

Written informed consent was obtained from every patient, or a caregiver in instances where the patient was unable to give consent before the study.

## Results

**Figure 1:**
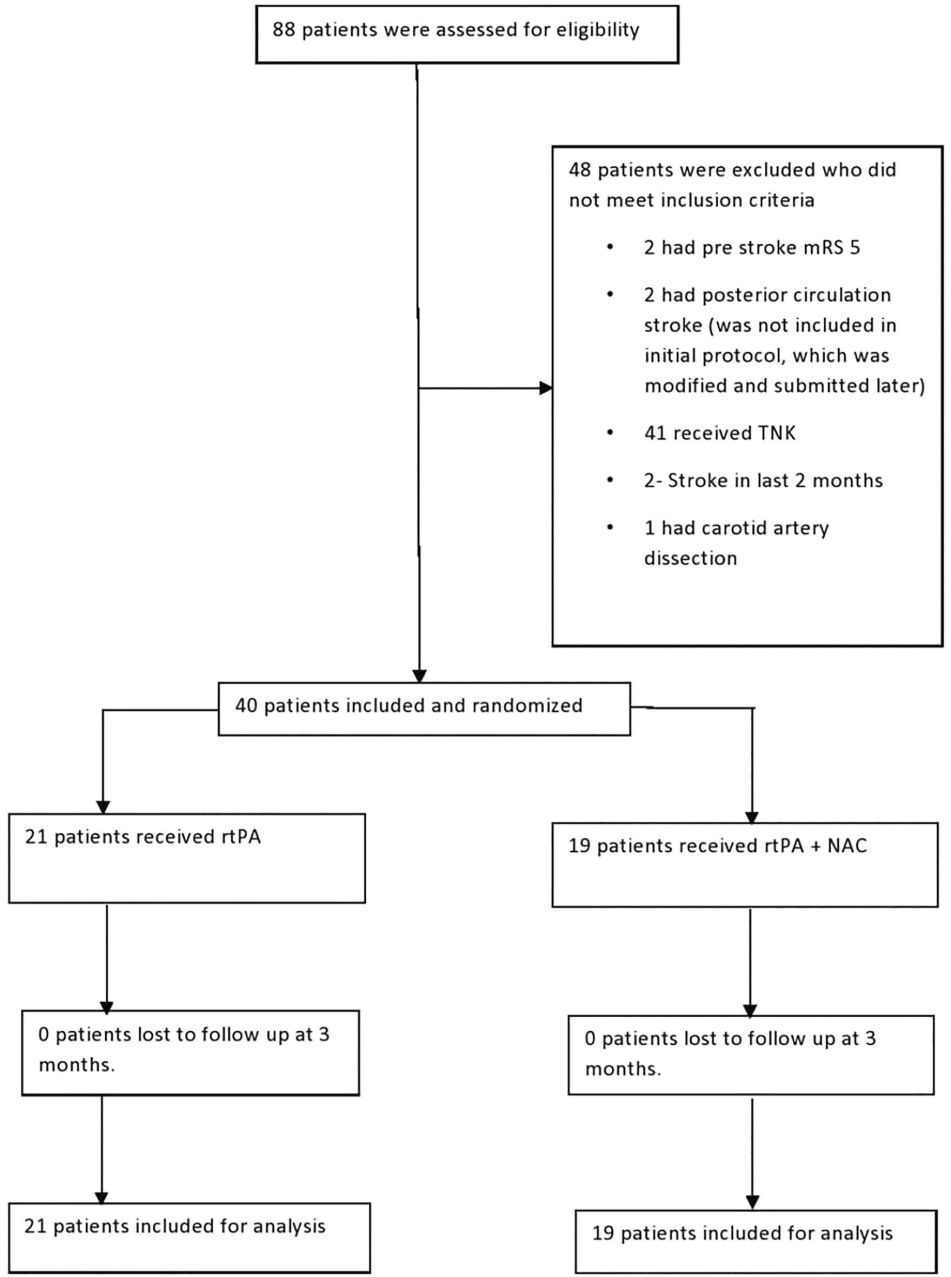
CONSORT Flow chart.

A total of 88 patients were screened for eligibility and 40 patients were included in the study with 21 in the control arm and 19 in the intervention arm. No patient was lost to follow up. The flow chart for the study is depicted in Fig. 1.

### Baseline characteristics

The baseline characteristics of the patients in the intervention and control groups are outlined in Table 2. The mean age (SD) was 54.09 (15.3) in the control arm and 51.36 (15.5) in the intervention arm. Majority of the patients were males; (12 [57.14%]) in the control group and 12 (68.42%) in the intervention group.

**Table 2:** Comparison of baseline characteristics, vascular risk factors, clinical and imaging characteristics.

Total (N = 40)	TPA (N = 21)	TPA + NAC (N = 19)	*P* value
Age (years)	54.09 ± 15.3	51.36 ± 15.5	0.57
Sex			
Male	12 (57.14%)	13 (68.42%)	0.52
Female	9 (42.86%)	6 (31.58%)	
Pre stroke mRS			
0	20 (95.24%)	17 (89.47%)	0.59
1	1 (4.76%)	2 (10.53%)	
SBP*	145.42 ± 30.42	140.57 ± 29.2	0.61
DBP*	84.09 ± 12.46	87.57 ± 10.25	0.34
Prior stroke	3 (14.29%)	4 (21.05%)	0.68
Hypertension	11(52.38%)	9 (47.36%)	0.75
Diabetes	6 (28.57%)	4 (21.05%)	0.72
Dyslipidemia	4 (19.05%)	4 (21.05%)	0.99
Coronary artery disease	6 (28.57%)	4 (21.05%)	0.72
Non-valvular atrial fibrillation	3 (14.28%)	3 (15.78%)	0.99
Rheumatic heart disease	2 (9.52%)	0	0.48
Chronic kidney disease	1 (4.76%)	1 (5.26%)	0.99
Smoking	5 (23.81%)	7 (36.84%)	0.49
Alcohol	2 (9.52%)	4 (21.05%)	0.39
Use of antiplatelets			
Aspirin 75 mg	5 (83.3%)	4 (57.1%)	0.73
Aspirin 150 mg	1 (16.7%)	1 (14.2%)	
Aspirin 75 mg + Clopidogrel 75 mg	0 (N=6)	2 (28.6%) (N=7)	
Use of oral anticoagulants			
Acitrom	1 (25%)	0	0.10
Warfarin	2 (50%)	0	
Dabigatran	1 (25%) (N=4)	0 (N=0)	
NIHSS median (IQR)	8 (5–13)	9 (6–14)	0.72
Vascular territory			
MCA	21 (100%)	15 (78.95%)	0.04
ACA	0	2 (10.53%)	
MCA + ACA	0	1 (5.26%)	
PCA	0	1 (5.26%)	
ASPECTS median (IQR)	8 (6–10)	9 (8–10)	0.21
Acute vascular imaging			
M1 occlusion	7 (87.5%)	3 (42.8%)	0.31
M2 occlusion	1 (12.5%)	3 (42.8%)	
ICA + MCA occlusion	0 (N = 8)	1 (14.28%) (N = 7)	
TOAST classification			
LAD EC	0	1 (5.26%)	0.22
LAD IC	4 (19.05%)	5 (26.32%)	
SVD	9 (42.86%)	5 (26.32%)	
CE	8 (38.10%)	5 (26.32%)	
Indeterminate	0	0	
Other determinate	0	3 (15.79%)	

*mRS* Modified Rankin Scale; *SBP* Systolic blood pressure; *DBP* Diastolic blood pressure. *NIHSS* National Institute of Health Stroke Scale; *ASPECTS* Alberta stroke program early CT score; *TOAST* Trial of Org 10172 in acute ischemic stroke; *MCA* Middle cerebral artery; *ACA* Anterior cerebral artery; *ICA* Internal carotid artery; *M1*: sphenoidal or horizontal segment of middle cerebral artery; *M2*: insular segment of middle cerebral artery; *LAD EC* large artery disease extracranial; *LAD IC* large artery disease intracranial; *SVD* small vessel disease; *CE* cardioembolism.

*Blood pressure in mm of Hg.

Baseline blood pressure measurements were similar between both study groups. Hypertension (20 [50%]) was the most common risk factor followed by tobacco use (12 [30%]), CAD (10 [25%]), diabetes mellitus (10 [25%]), dyslipidemia (8 [20%]), prior stroke (7 [17.5%]), alcohol abuse (6 [15%]), non valvular atrial fibrillation (NVAF) (6 [15%]), RHD (2 [5%]) and CKD (2 [5%]) (Table 2). Two patients had pacemakers, one patient had pemphigus vulgaris, one had history of renal transplant, one had ulcerative colitis and one had tubercular arachnoiditis. One patient was People living with HIV/AIDS (PLWHA), and one patient was COVID positive. There was no significant difference between two groups for these variables. Of the participants, 32.5% were on prior antiplatelet therapy, and this distribution was comparable between the intervention and control groups. In the control group, four patients reported prior use of oral anticoagulants, while none in the intervention group had a history of such use.

Detailed baseline clinical and imaging characteristics are provided in Table 2. The baseline NIHSS scores were comparable, with medians of 8 (IQR: 5–13) and 9 (IQR: 6–14) in the control and intervention groups, respectively. Similarly, the ASPECTS at presentation did not show a significant difference between the two groups, with medians of 8 (IQR: 6–10) and 9 (IQR: 8–10) in the control and intervention groups, respectively. MCA territory infarcts were prevalent, constituting 90% of cases.

Vascular imaging performed at admission revealed occlusion of the large vessels in 15 patients (37.5%), evenly distributed across both groups. The major etiology for ischemic stroke was small vessel disease (35%), followed by cardioembolic causes (32.5%), large artery disease (25%), and other determinate causes (7.5%).

In the intervention arm, two patients (10.52%) experienced in-hospital strokes. Key time metrics for thrombolysis, encompassing onset to door time, onset to TPA time, door to TPA time, and door to CT time, are visually represented in box plots. The disparity in the median onset to door time between the two groups did not reach statistical significance (*p* = 0.77) (Fig. S 1). Similarly, no significant variance in door to CT time was detected between the two groups (*p* = 0.27) (Fig. S 2). The onset to TPA duration demonstrated comparable profiles in both groups (Fig. S 3). The median (IQR) door to TPA duration in the control arm was 30 (20–30) minutes, while in the intervention arm, it was 20 (20–30) minutes (*p* = 0.62) (Fig. S 4). The intervention group demonstrated a median (IQR) onset to *N*-acetylcysteine (NAC) time of 160 (100–215) minutes (N = 19), while the door to NAC time was 25 (25–45) minutes (N = 17) (Table S1).

Ten patients (66.66%) underwent thrombectomy, with seven (33.33%) in the control group and four (21.05%) in the intervention group (*p* = 0.48, see Table S2). Notably, 90.9% of patients achieved recanalization, while one patient experienced failed recanalization. All patients in the intervention arm attained mTICI 3 recanalization, although this distinction did not achieve statistical significance (*p* = 0.53). Box plots depicting time metrics for the endovascular procedure, such as onset to groin puncture and door to groin puncture, demonstrate similar timelines in both groups (Figs. S 5 and S 6, respectively). The median (IQR) time from groin puncture to recanalization was 20 (17.5–20) minutes in the intervention arm compared to 15 (15–15) minutes in the control arm (*p* = 0.94).

### Outcomes primary outcomes

*Adverse Reactions* Moderate adverse reactions were reported in 5% of the cohort. Within the control arm, a single patient (4.76%) exhibited transient vomiting, promptly ameliorated with intravenous antiemetic therapy. Conversely, a participant in the intervention arm (5.26%) reported pruritus, devoid of cutaneous manifestations or erythema, six hours post-infusion. This resolved satisfactorily following administration of an oral antihistamine. Remarkably, no instances of life-threatening or serious adverse events were documented*.*

*Intracerebral hemorrhage* Intracerebral hemorrhage occurred in two patients (10.53%) in the intervention arm, while none were observed in the control arm (95% CI − 24.3 to 3.27; *p* = 0.21). One patient in the intervention arm experienced symptomatic intracerebral hemorrhage. Statistical significance remained elusive for this discrepancy (95% CI − 15.30 to 4.77; *p* = 0.47) (Table 3).

**Table 3:** Comparison of primary outcomes.

Total (N = 40)	TPA (N = 21)	TPA + NAC (N = 19)	*P* value	Effect size (95% CI)
Any ICH (including SICH)	0	2 (10.53%)	0.21	− 10.5 (− 24.3 to 3.27)
SICH	0	1 (5.26%)	0.47	− 5.1 (− 15.3 to 4.77)
Extracranial bleeding				
Mild	0	2 (10.53%)	0.21	− 10.5 (− 24.3 to 3.27)
Moderate	0	0		
Major	0	0		
Adverse reactions				
Vomiting	1 (4.76%)	0	0.99	− 5.2(− 15.3 to 4.77)
Itching	0	1 (5.26%)		

*ICH* Intracerebral Hemorrhage; *SICH* Symptomatic intracerebral hemorrhage.

*Extracranial bleeding* In the intervention arm, a subset of patients (10.53%) reported occurrence of mild extracranial bleeding. These included a 1–2 ml ear bleed in one case and a streak of gum bleeding in another, both of which resolved spontaneously. Intriguingly, this observed variance failed to attain statistical significance (95% CI − 24.3 to 3.27; *p* = 0.21) (Table 3). Notably, no instances of major extracranial bleeding were documented.

### Secondary Outcomes

*Major neurological improvement at 24 h* A notable cohort of 11 patients (27.5%) exhibited a substantial improvement in the NIHSS, with a reduction of ≥ 8 points from baseline or reaching NIHSS 0. Of this group, 42.1% (8 patients) belonged to the intervention arm, in contrast to 14.28% (3 patients) in the control arm (95% CI − 54.59 to 1.04; *p* = 0.10) (Table 4). Patients in the intervention group demonstrated significantly lower NIHSS scores at 24 h compared to those in the control arm (*p* = 0.03), as detailed in Table 4.

**Table 4:** Comparison of secondary outcomes.

Total (N = 40)	TPA (N = 21)	TPA + NAC (N = 19)	*P* value	Effect size (95% CI)
NIHSS at 24 h Median (IQR)	8 (3–14)	2 (0–6)	0.03	NA
Major neurological improvement at 24 h	3 (14.28%)	8 (42.1%)	0.10	− 27.8 (− 54.59 to 1.04)
Recanalization on repeat vascular imaging after 24 h	0 N = 1	2 (50%) N = 4	0.99	NA
mRS at 3 months				
0–2 (good outcome)	16 (76.19%)	14 (73.68%)	0.85	− 2.5 (− 24.3 to 29.4)*
3–6 (poor outcome)	5 (23.8%)	5 (26.31%)		
Mortality at 3 months	2 (9.52%) N = 21	2 (10.53%) N = 19	0.99	− 1.0 (− 19.6 to 17.6)

*NA* Not applicable.

*Effect size and 95% Confidence Intervals mentioned for good outcome (mRS 0–2) at 3 months.

*Recanalization at angiography during thrombectomy or at 24 h* No instances of recanalization were observed during the initial angiography run for patients undergoing thrombectomy. At the 24-h, five patients (13.1%) underwent repeat vascular imaging (TOF MRA); one in the control arm and four in the intervention arm. Intriguingly, 50% (2 patients) in the intervention arm achieved a mTICI 3 recanalization, while none in the control arm achieved the same (Table 4). The difference observed was not significant (*p* = 0.99).

**Figure 2:**
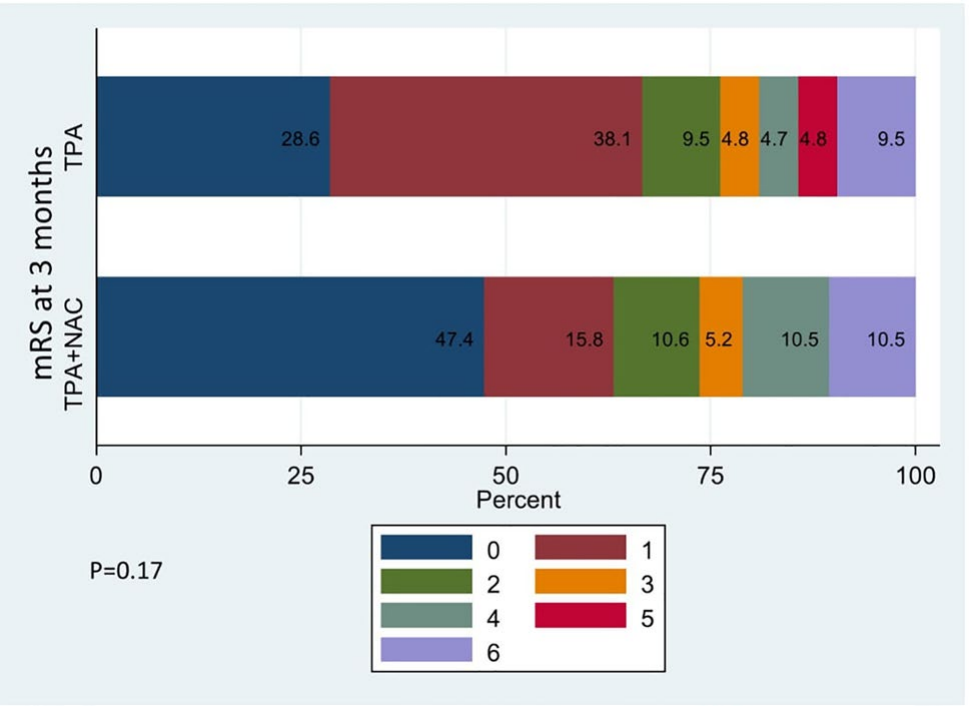
Distribution of mRS 0–6 at 3 months among both the groups.

*Morbidity and mortality at 3 months* The proportion of patients attaining a positive outcome was comparable between the intervention and control groups, with 73.68% (14 patients) and 76.19% (16 patients), respectively (95% CI − 24.3 to 29.4; *p* = 0.85, Table 4). Mortality at 3 months demonstrated similarity between the two groups (95% CI − 19.6 to 17.6; *p* = 0.99). Although no statistically significant variance surfaced in the distribution of mRS scores between the groups, it is of note that patients achieving an mRS score of 0 at 3 months exhibited a numerical preponderance in the intervention arm (*p* = 0.17, Fig. 2).

**Figure 3:**
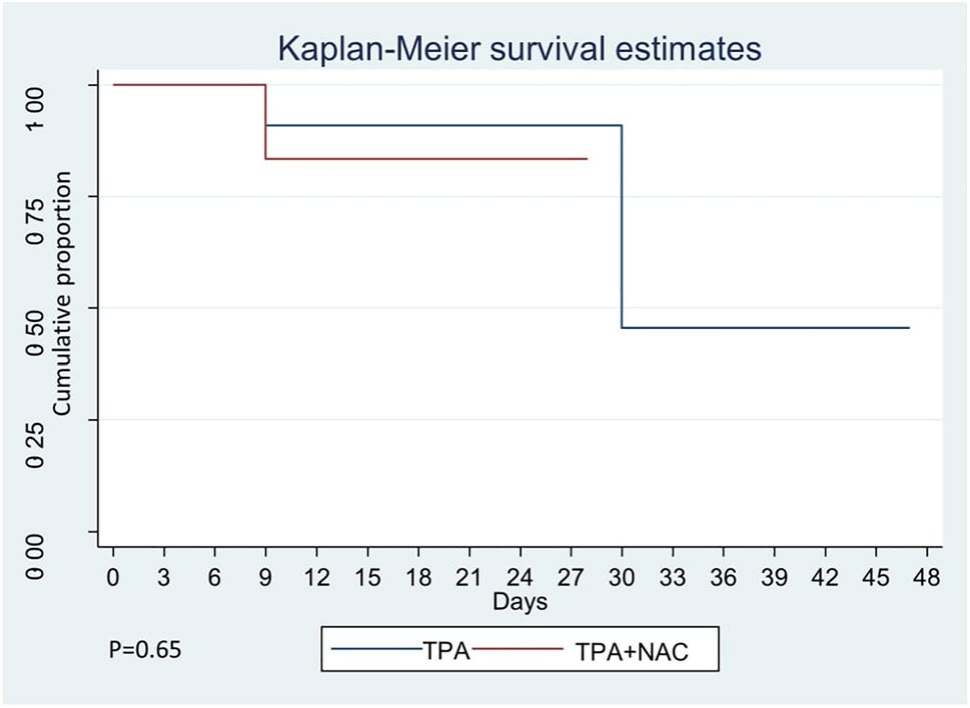
Comparison of survival analysis among both groups.

We conducted a comprehensive assessment of the survival function for both study groups, illustrated in Fig. 3. In the control group, the median survival was 30 days. However, in the intervention group, presenting the median survival proved challenging, as the cumulative survival exceeded 50% after a 3-month follow-up. Additionally, determining the 70% or 75% survival in the intervention group was not feasible. The contrast in survival between the control arm and the intervention group did not reach statistical significance (*p* = 0.65).

The duration of hospitalization was similar between both groups (*p* = 0.30) (Table S 3). Infections during hospitalization were documented in seven patients (17.5%). The predominant infection type was ventilator-associated pneumonia in four cases (57.14%), followed by bloodstream infection in two cases (28.57%), and hospital-acquired pneumonia in one case (14.28%). Ventilatory assistance was required by five patients (12.5%), of whom three needed prolonged ventilatory support and underwent tracheostomy. In-hospital mortality did not exhibit a significant difference between the two groups (*p* = 0.92) (Table S 3). Importantly, none of the patients underwent neurosurgical procedures during their hospital stay.

## Discussion

Current study is the first randomized control trial in humans, exploring the safety and efficacy of *N*-acetylcysteine as an add on thrombolytic agent to alteplase for acute ischemic stroke. Our observations revealed that patients administered *N*-acetylcysteine alongside alteplase exhibited more favorable early neurological improvement. Notably, there were no significant disparities in disability at three months, overall good outcomes, or mortality at three months between the two groups. Furthermore, adverse events did not demonstrate notable differences with the addition of *N*-acetylcysteine to alteplase when compared to alteplase alone. Baseline characteristics were comparable among both groups. In our study population, the etiology of ischemic stroke differed among two groups. Although this difference is not significant, this imbalance might be relevant in interpreting our neutral result of disability outcome at three months. The study population in our trial differed from those in previous trials of alteplase with respect to comorbidities reflecting wider use of alteplase in clinical practice at the time of our study.

Early neurological improvement within the initial 24 h post-stroke is an independent predictor of favorable outcomes at 12 months^12^. While the functional outcome at three months is not solely contingent on recanalization within the first 24 h of thrombolytic therapy, it may be influenced by factors such as collateral blood supply or retrograde flow. In our investigation, patients receiving *N*-acetylcysteine with alteplase exhibited a significantly lower median NIHSS at 24 h. Within this subset, early neurological improvement correlated with a favorable outcome at three months, potentially attributed to improved intracranial arterial recanalization facilitated by the addition of *N*-acetylcysteine. It is noteworthy that the half-life of *N*-acetylcysteine is brief, and its lytic activity can persist up to 12 h post-infusion^13^. Consequently, early reperfusion, best evidenced at 24 h, serves as a reliable measure of early neurological improvement^14^.

Our study also sought to assess recanalization at the initial angiogram run in the enrolled patients. While recanalization serves as a robust predictor of outcomes, the timing of recanalization and reperfusion, particularly early occurrences, holds greater relevance than late^15^. However, the predefined endpoint of achieving recanalization at the first run of angiography was not met by any of the patients. The constrained sample size likely exacerbated the inherent statistical challenge of identifying subtle treatment benefits associated with N-acetylcysteine, especially given the well-documented low recanalization rates of alteplase (TPA) alone in large vessel occlusion (LVO) cases^2^.

Despite a small number undergoing endovascular therapy and a limited number of patients undergoing repeat vascular imaging at 24 h, noteworthy recanalization at 24 h favored those who received *N*-acetylcysteine with alteplase. While challenging to definitively establish, this observation may be linked to the nature of the arterial thrombus. It underscores the crucial consideration that the composition of the clot could play a pivotal role in determining the efficacy of the thrombolytic agent employed. The thrombolytic effect of *N*-acetylcysteine is anticipated to primarily involve the cleavage of von Willebrand factor (VWF), which cross-links platelets within arterial thrombi, often originating from atherosclerotic lesions^7^. This recognition underscores the importance of understanding clot composition in tailoring thrombolytic strategies for optimized outcomes.

The study employed an FDA-approved dose of 150 mg/kg of *N*-acetylcysteine, a regimen established for acetaminophen overdose treatment. Our observed adverse event profile closely aligned with published data regarding incidence and severity, validating external generalizability and suggesting predictable and manageable risks in future applications^16^.

The study's strengths encompass its randomized controlled design, concealed allocation, and blinded assessment with complete follow-up. However, limitations include a reduced sample size due to the ongoing pandemic's impact on admissions. The study exclusively employed alteplase as a comparator to mitigate heterogeneity, given the frequent use of Tenecteplase (TNK) at our center. Subgroup analyses were precluded by the small patient cohort, and the open-label design may introduce potential bias, especially in acute phase outcome measurement.

## Conclusion

The addition of *N*-acetylcysteine to alteplase did not yield a significant difference in morbidity and mortality at the 3 months. *N*-acetylcysteine exhibited a safety profile akin to that of alteplase. Despite these comparable outcomes, there was a noteworthy trend favoring *N*-acetylcysteine in terms of early neurological improvement within the initial 24 h. Given these findings, future investigations with a larger sample size are warranted to comprehensively assess the safety and efficacy of *N*-acetylcysteine as a thrombolytic agent.

### Supplementary Information

Supplementary Information.

## Data Availability

The datasets used and analyzed during the current study available from the corresponding author (RB) on reasonable request.
