# Supplementary material for: Safety and efficacy of N-acetylcysteine (NAC) as an adjunct to standard treatment in patients with acute ischemic stroke: a randomized controlled pilot trial (NACTLYS)
Source: Sci Rep. 2024 Jan 11;14:1103. doi: 10.1038/s41598-023-49054-9 (PMC10784280; doi:10.1038/s41598-023-49054-9)

**Supplemental Material** Supplemental Literature Review Supplemental Operational Definitions Tables S1–S4

Figure S1-S6

**Supplemental Literature Review**

**N Acetyl cysteine – Pharmacology, uses and adverse effects:**

N-acetyl Cysteine (NAC) is acetyl derivative of the amino acid cysteine[1]. It also has antioxidant and a free-radical scavenging activity that increases intracellular Glutathione (GSH), a major component of thepathways by which cells are protected from oxidative stress[2]. Low bioavailability of NAC is oneof the major limitations for maximizing its effects on oxidative stress-related diseases. NAC is considered a well-tolerated and safe medication that has been used all across the world in varietyof medical conditions for past several decades. It is widely recognized for its role as an antidote in acetaminophen overdose and has been approved by the FDA for the same[3]. It is also used as amucolytic in chronic obstructive pulmonary disease[4], a renal protectant in contrast-induced nephropathy[5], as a preventive agent for atrial fibrillation[6] and as adjunct therapy in HIV infection[7]. Additionally, it has shown to inhibit replication of the seasonal influenza A virus andcould be a potential treatment in influenza A infection[8].

With oral intake of NAC at the dose of 200–400 mg, the peak plasma concentration of 0.35–4 mg/L is achieved within 1–2h after ingestion. Information on interaction with food is lacking. The volume of distribution ranges from 0.33 to 0.47 L/kg and protein binding is signiﬁcant being 50% at 4 h after the dose administration. Intravenously infused NAC rapidly forms disulﬁdes in plasma, which prolong the existence of the drug in plasma for up to 6 hours[9].

Renal clearance has been reported at 0.190–0.211 L/h per kg; however, up to 70% of the total body clearance is nonrenal. NAC is generallysafe and well tolerated even at high doses. There are reports of adverse drug reactions (ADR) toIV-NAC with incidence ranging from 3% to 77%. ADR to IV- NAC is well described and commonly occur during the loading-dose infusion. They are thought to be dose dependent (e.g.,rate of infusion) and anaphylactoid in origin[10-13].

Most of ADR typically occur within the first hour of commencement of NAC infusion. These reactions have been characterized by the following features: nausea, vomiting, flushing, rash, pruritis, headache, dizziness, chest pain, coughing, and convulsion. More serious ADR including bronchospasm and hypotension have also been reported in low frequency. Acetylcysteine has been associated with worsening asthmatic symptoms[14]. Overall, ADR to IV-NAC is usually mildand often symptoms will resolve on temporary stop of the NAC infusion. If required, corticosteroids, antihistamines, inhaled beta-agonists, and in severe cases intramuscular adrenaline have all been successfully used to bring about symptomatic improvement and allow continuation of NAC infusion[15,16].

NAC strongly potentiates the effect of nitroglycerin and related medications, and caution should be used in patients receiving these agents in whom it may cause hypotension[9]. Other miscellaneous AEs reported were fatigue, dry mouth, muscle pains, insomnia, nasal congestion, runny nose, restlessness, and dizziness and vivid dreams and irritability[17,18]. Neurological side effect commonly reported was headache[19]. Two rare adverse events were reported - neutropenia with 6 g of NAC that improved once the dose was dropped down to 2.4 g daily and irreversible sensorineural deafness leading to discontinuation of NAC at 5 weeks[20]. These were on prolongedoral route of administration.

# Evidence of N acetyl cysteine Efficacy in Ischemic stroke:

Arterial thrombi often originate from atherosclerotic lesions, secondary to high shear stress rates. High shear arterial thrombosis involves mainly von Willebrand Factor (VWF)-dependent platelet cross-linking[21]. Thus, proteolysis of VWF could be an efficient strategy to disaggregate arterial and platelet-rich thrombi. Thrombolytic effect of NAC administration is mainly mediated by cleavage of the VWF that crosslinks platelets inside arterial thrombi[22].

Martinez and colleagues created two mouse models with FeCl3-induced platelet-rich thrombi and thrombin induced formation of mixed thrombi, in the middle cerebral artery (MCA) with

<20% decrease in cerebral blood flow which was determined by laser Doppler flowmetry. In FeCl3 model, 400 mg/kg of NAC injected as a slow intravenous bolus (60 seconds) 20 minutes after occlusive thrombus formation. NAC administration led to a rapid and significant reperfusion reaching up to 53% of the baseline cerebral blood flow (CBF), as measured by laser Doppler flowmetry. This reperfusion was transient and followed by rapid rethrombosis. Whereas tPA 10mg/kg (10% bolus, 90% infusion over 40 minutes) given 20 minutes after thrombus formation failed to influence the lesion size in the FeCl3 model. This data suggest that the thrombolytic effects of N acetyl cysteine are independent of plasmin generation, anticoagulation, or platelet activation inhibition[23].

They injected NAC 20 minutes after ischemic onset in 3 different stroke models: electrocoagulation (permanent ischemia), thrombin injection (mixed thrombi with significant amount of VWF) and topical FeCl3 application (platelet-rich thrombi). NAC significantly reduced ischemic lesion sizes in both thrombin-induced (–39%, P<0.05) and FeCl3-induced (– 57%, P<0.05) stroke models as assessed after 24 hours using MRI scan. In contrast, NAC failed to reduce the ischemic lesion size in the electrocoagulation model (–10%, P=0.57), suggesting

that its brain-protective effects in our experimental conditions are related to arterial recanalization rather than direct neuroprotection. These results demonstrate that NAC administration is beneficial in acute ischemic stroke because of its thrombolytic properties. Overall, these results demonstrate that NAC acts as a thrombolytic in the presence of platelet rich (FeCl3 model) and mixed thrombi (thrombin model) and improves ischemic stroke outcome when injected as a monotherapy[23].

Sekhon and colleagues created a rat model where focal cerebral ischemia produced by middle cerebral artery occlusion and pretreated with NAC at150 mg/kg, showed a 49.7% reduction in brain infarct volume and 50% reduction in the neurological evaluation score compared to untreated animals. Also decreased the TNF-a expression and iNOS expression on immunohistochemistry[24].

In animal study by S. Cuzzocrea et al, N Acetyl Cysteine (20 mg /kg) was given as an intraperitoneal bolus 30 min before reperfusion and and 1, 2 and 6 h after reperfusion) reduced the formation of post-ischemic brain edema, evaluated by water content. NAC also attenuated the increase in the hippocampus of myeloperoxidase (MPO) caused by cerebral ischemia. In addition, NAC reduces the recruitment of polymorphonuclear leukocytes (PMNs) into the inflammatory site. NAC scavenges and inactivates superoxide anions and nitric oxide. These results showed that NAC improves brain injury induced by transient cerebral ischemia[25].

# N Acetyl cysteine Safety Profile in Hemorrhagic stroke models:

In the study by Martinez and colleagues, a hemorrhagic stroke model was created, where mice were subjected to intracranial hemorrhage induced by intra-striatal administration of 0.1 UI type

VII collagenase Unlike intravenous heparin administration (200 UI/kg in bolus, 75 minutes after collagenase administration) that led to hematoma expansion and a high mortality rate, N Acetyl Cysteine (NAC) treatment (400 mg/ kg in bolus, 75 minutes after collagenase administration) failed to promote hematoma expansion, worsen clinical score, or increase lesion sizes at any time points in comparison with saline-treated animals. Hence, NAC displayed a favorable safety profile even in hemorrhagic stroke model[23].

Guney and colleagues studied N Acetyl cysteine in subarachnoid hemorrhage by intraperitoneal injection in rabbits and found it was effective against cerebral vasospasm following SAH. Also, NAC treatment increased the luminal area and reduced wall thickness of the basilar artery[26].

Thus N-Acetyl cysteine has been studied in many animal models, both in safety and efficacy in stroke models both ischemic and hemorrhagic. N Acetyl cysteine is being used in many conditions already and its safety is proven in human studies. Hence, Phase II human trial to study its safety profile and efficacy as thrombolytic agent along with alteplase was considered.

**Supplemental Operational Definitions**

Definition of Adverse Event[15]:

- “Adverse event” was defined as any anaphylactoid (cutaneous or systemic) or life- threatening event occurring after the initiation of oral N-acetylcysteine by the intravenous route.
- “Cutaneous” was defined as itching, rash, flushing, urticaria, or similar symptoms without other effects. “Systemic” was defined as any respiratory or cardiovascular effects such as wheezing, dyspnea, or transient hypotension not requiring intervention.
- “Life threatening” was defined as cardiac arrest, cardiac instability, respiratory arrest, or

hypotension requiring intervention such as pressors.

Adverse events[20]:

1. *Minimal:* if patients had no reaction or mild gastrointestinal symptoms only and does not require any specific treatment
2. *Moderate*: mild flushing, pruritus, mild chest pain, breathlessness, gastrointestinal symptoms like nausea/vomiting and requires temporary cessation of NAC infusion and requires administration of antiemetics, antihistamines, corticosteroids or selective beta 2- adrenoreceptoragonists.
3. *Severe*: Severe flushing, respiratory distress, angioedema, moderate to severe chest pain, hypotension (systolic blood pressure < 90 mmHg or diastolic blood pressure <50 mmHg) which requires stoppage of NAC infusion and initiation of symptomatic treatment.

ICH Definitions as per SITS MOST criteria [27]:

*Hemorrhagic infarction type 1 (HI1):* small petechiae along the margins of the infarct. *Hemorrhagic infarction type 2 (HI2):* confluent petechiae within the infarcted area without space-occupying effect.

*Parenchymal hemorrhage type 1 (PH1):* local, or intra-ischemic confluent hematoma in ≤ 30% of the infarcted area with at the most some slight space-occupying effect.

*Parenchymal hemorrhage type 2 (PH2):* local, or intra-ischemic confluent hematoma >30% of the infarcted area with a substantial space-occupying effect.

*Remote parenchymal hemorrhage type 1 (PHr1):* small to medium sized hematoma located remote from the infarct(s), with mild space occupying effect.

*Remote parenchymal hemorrhage type 2 (PHr2):* large confluent hematoma in an area remote from the actual infarct(s), with substantial space occupying effect.

SICH (Symptomatic Intracranial Hemorrahge) Definition[27]:

SICH per SITS-MOST(Safe Implementation of Treatment in Stroke- Monitoring study): Local or remote parenchymal haemorrhage type 2 on the 22-36 h post-treatment imaging scan, combined with a neurologic deterioration of 4 points or more compared to baseline NIHSS or the lowest NIHSS value between baseline and 24 h or death within 24 h. Type 2 indicates a hematoma exceeding 30% of the infarct, with substantial space-occupying effect.

Major Bleeding :ISTH ( International Society on Thrombosis and Haemostasis)[28] defined major

bleeding as having a symptomatic presentation and i Fatal bleeding, and/or

ii Bleeding in a critical area or organ, such as intracranial, intraspinal, intraocular, retroperitoneal, intraarticular or pericardial, or intramuscular with compartment syndrome, and/or

iiiSignificant extracranial haemorrhage (requirement for blood transfusion or drop in haemoglobin of ≥20mg/l in the 36h after treatment)

Minor Bleeding: Any sign or symptom of hemorrhage (e.g., more bleeding than would be

expected for a clinical circumstance, including bleeding found by imaging alone) that does not

fit the criteria for the ISTH definition of major bleeding but does meet at least one of the following criteria:

i requiring medical intervention by a healthcare professionalii leading to hospitalization or increased level of care

iii prompting a face to face (i.e., not just a telephone or electronic communication) evaluation.

Modified treatment in cerebral infarction (mTICI) score[29]:

Grade 0: no perfusion

Grade 1: Antegrade reperfusion past the initial occlusion, but limited distal branch fillingwith little or slow distal reperfusion

Grade 2

Grade 2a: Antegrade reperfusion of less than half of the occluded target artery previouslyischemic territory (e.g. in one major division of the middle cerebral artery (MCA) and itsterritory)

Grade 2b: Antegrade reperfusion of more than half of the previously occluded target arteryischemic territory (e.g. in two major divisions of the MCA and their territories)

Grade 3: Complete antegrade reperfusion of the previously occluded target artery ischemicterritory, with absence of visualized occlusion in all distal branches.

# References

1. Arakawa M, Ito Y. N-acetylcysteine and neurodegenerative diseases: basic and clinical pharmacology. Cerebellum Lond Engl. 2007;6(4):308–14.
2. Berk M, Malhi GS, Gray LJ, Dean OM. The promise of N-acetylcysteine in neuropsychiatry. Trends Pharmacol Sci. 2013 Mar;34(3):167–77.
3. Yarema MC, Johnson DW, Berlin RJ, Sivilotti MLA, Nettel-Aguirre A, Brant RF, et al.

Comparison of the 20-Hour Intravenous and 72-Hour Oral Acetylcysteine Protocols for the Treatment of Acute Acetaminophen Poisoning. Ann Emerg Med. 2009 Oct;54(4):606–14.

1. Dekhuijzen P, van Beurden W. The role for N-acetylcysteine in the management of COPD. Int J COPD. 2006 Apr;1(2):99–106.
2. Briguori C, Donnarumma E, Quintavalle C, Fiore D, Condorelli G. Contrast-induced acute kidney injury: potential new strategies. Curr Opin Nephrol Hypertens. 2015 Mar;24(2):145– 53.
3. Liu X-H, Xu C-Y, Fan G-H. Efficacy of N-acetylcysteine in preventing atrial fibrillation after cardiac surgery: a meta-analysis of published randomized controlled trials. BMC Cardiovasc Disord [Internet]. 2014 Dec [cited 2019 Jan 12];14(1). Available from:<http://bmccardiovascdisord.biomedcentral.com/articles/10.1186/1471->2261-14-52.
4. De Rosa SC, Zaretsky MD, Dubs JG, Roederer M, Anderson M, Green A, et al. N- acetylcysteine replenishes glutathione in HIV infection. Eur J Clin Invest. 2000 Oct;30(10):91529.
5. Geiler J, Michaelis M, Naczk P, Leutz A, Langer K, Doerr H-W, et al. N-acetyl-l- cysteine (NAC) inhibits virus replication and expression of pro-inflammatory molecules in A549 cells infected with highly pathogenic H5N1 influenza A virus. Biochem Pharmacol. 2010 Feb;79(3):413–20.
6. Bavarsad Shahripour R, Harrigan MR, Alexandrov AV. N -acetylcysteine (NAC) in neurological disorders: mechanisms of action and therapeutic opportunities. Brain Behav. 2014 Mar;4(2):108–22.
7. Schmidt LE, Dalhoff K. Risk factors in the development of adverse reactions to N- acetylcysteine in patients with paracetamol poisoning. 2001;5.
8. Kao LW, Kirk MA, Furbee RB, Mehta NH, Skinner JR, Brizendine EJ. What is the rate of adverse events after oral N-acetylcysteine administered by the intravenous route to patients with suspected acetaminophen poisoning? Ann Emerg Med. 2003 Dec;42(6):741–50.
9. Merl W, Koutsogiannis Z, Kerr D, Kelly A. How Safe is Intravenous N- Acetylcysteine for the Treatment of Paracetamol Poisoning? Hong Kong J Emerg Med. 2007 Oct;14(4):198– 203.
10. Lynch RM, Robertson R. Anaphylactoid reactions to intravenous N-acetylcysteine: a prospective case controlled study. Accid Emerg Nurs. 2004 Jan;12(1):10–5.
11. Ho SW, Beilin LJ. Asthma associated with N-acetylcysteine infusion and paracetamol poisoning: report of two cases. BMJ. 1983 Sep 24;287(6396):876–7.
12. Pakravan N, Waring WS, Sharma S, Ludlam C, Megson I, Bateman DN. Risk factors and mechanisms of anaphylactoid reactions to acetylcysteine in acetaminophen overdose. Clin Toxicol. 2008 Jan;46(8):697–702.
13. Waring WS, Stephen AF, Robinson OD, Dow MA, Pettie JM. Lower incidence of anaphylactoid reactions to N-acetylcysteine in patients with high acetaminophen concentrations after overdose. Clin Toxicol. 2008 Jan;46(6):496–500.
14. Grant JE, Odlaug BL, Chamberlain SR, Potenza MN, Schreiber LRN, Donahue CB, et al.

A Randomized, Placebo-Controlled Trial of N -Acetylcysteine Plus Imaginal Desensitization for Nicotine-Dependent Pathological Gamblers. J Clin Psychiatry. 2014 Jan 15;75(01):39– 45.

1. Gray KM, Carpenter MJ, Baker NL, DeSantis SM, Kryway E, Hartwell KJ, et al. A Double- Blind Randomized Controlled Trial of N -Acetylcysteine in Cannabis- Dependent Adolescents. Am J Psychiatry. 2012 Aug;169(8):805–12.
2. LaRowe SD, Kalivas PW, Nicholas JS, Randall PK, Mardikian PN, Malcolm RJ. A double- blind placebo-controlled trial of N-acetylcysteine in the treatment ofcocaine dependence: N - Acetylcysteine for Cocaine Dependence. Am J Addict. 2013 Sep;22(5):443–52.
3. Edwards MJJ, Hargreaves IP, Heales SJR, Jones SJ, Ramachandran V, Bhatia KP, et al.

N- acetylcysteine and Unverricht-Lundborg disease Variable response and possible side effects. Neurology. 2002 Nov 12;59(9):1447–9.

1. Le Behot A, Gauberti M, Martinez De Lizarrondo S, Montagne A, Lemarchand E, Repesse Y, et al. GpIb -VWF blockade restores vessel patency by dissolving platelet aggregates formed under very high shear rate in mice. Blood. 2014 May 22;123(21):3354–63.
2. Nesbitt WS, Westein E, Tovar-Lopez FJ, Tolouei E, Mitchell A, Fu J, et al. A shear gradient–dependent platelet aggregation mechanism drives thrombus formation. Nat Med. 2009 Jun;15(6):665–73.
3. Martinez de Lizarrondo S, Gakuba C, Herbig BA, Repessé Y, Ali C, Denis CV, et al.

Potent Thrombolytic Effect of N -Acetylcysteine on Arterial Thrombi. Circulation. 2017 Aug 15;136(7):646–60.

1. Sekhon B, Sekhon C, Khan M, Patel SJ, Singh I, Singh AK. N-Acetyl cysteine protects against injury in a rat model of focal cerebral ischemia. Brain Res. 2003 May;971(1):1–8.
2. Cuzzocrea S, Mazzon E, Costantino G, Serraino I, Dugo L, CalabroÁ G, et al.

Benefecial effects of N-acetylcysteine on ischemic brain injury. Br J Pharmacol. 130:8.

1. Güney O, Erdi F, Esen H, Kiyici A, Kocaogullar Y. N-acetylcysteine prevents vasospasm after subarachnoid hemorrhage. World Neurosurg. 2010 Jan;73(1):42–9.
2. Wahlgren N, Ahmed N, Dávalos A, Ford GA, Grond M, Hacke W, et al. Thrombolysis with alteplase for acute ischaemic stroke in the Safe Implementation of Thrombolysis in Stroke- Monitoring Study (SITS-MOST): an observational study.2007;369:8.
3. Kaatz S, Ahmad D, Spyropoulos AC, Schulman S, the Subcommittee on Control of Anticoagulation. Definition of clinically relevant non-major bleeding in studies of anticoagulants in atrial fibrillation and venous thromboembolic disease in non-surgical patients: communication from the SSC of the ISTH. J Thromb Haemost. 2015 Nov;13(11):2119–26.
4. Coulthard A. Modified treatment in cerebral ischemia (mTICI) score | Radiology Reference Article | Radiopaedia.org [Internet]. Radiopaedia. [cited 2021 Feb 25]. Available from: https://radiopaedia.org/articles/modified-treatment-in-cerebral- ischaemia-mtici-score.

# Table S 1: Comparison of stroke time metrics of thrombolysis

| **Total (N=40)** | **TPA** | **TPA+NAC** | **P value** |
| --- | --- | --- | --- |
| **Onset to door time***  **Median (IQR)** | **120(120-180)**  **N=20** | **135(75-180)**  **N=17** | **0.77** |
| **Door to CT time***  **Median (IQR)** | **20(15-22.5)**  **N=20** | **15(10-20)**  **N=17** | **0.27** |
| **Onset to TPA time*** | **150(135-200)**  **N=21** | **140(95-210)**  **N=19** | **0.75** |
| **Onset to NAC time***  **Median (IQR)** | **-** | **160(100-215)**  **N=19** | **NA** |
| **Door to TPA time***  **Median (IQR)** | **30(20-30)**  **N=20** | **20(20-30)**  **N=17** | **0.62** |
| **Door to NAC time***  **Median (IQR)** | **-** | **25(25-45)**  **N=17** | **NA** |
| **Dose of TPA**  **received † Mean ±SD** | **59.84±10.44** | **60.76±12.48** | **0.80** |

***Duration in minutes. † dosage in milligrams(mg)**

# NA- Not applicable; NAC – N-acetylcysteine; TPA – Alteplase

**Table S 2: Comparison of time metrics of Endovascular Treatment**

| **Total (N=11)** | **TPA** | **TPA+NAC** | **P value** |
| --- | --- | --- | --- |
| **Endovascular Therapy** | **7 (33.33%)** | **4(21.05%)** | **0.48** |
| **Recanalization as per**  **mTICI grading* 0**  **2B**  **2C**  **3** | **1(14.29%)**  **2(28.57%)**  **1(14.29%)**  **3(42.86%) N=7** | **0**  **0**  **0**  **4(100%) N=4** | **0.53** |
| **Type of device Solitaire Solumbra** | **4(57.14%)**  **3(42.86%) N=7** | **3(75%)**  **1(25%) N=4** | **0.99** |
| **Onset to Groin**  **Puncture† Median (IQR)** | **228(90-250) N=7** | **210(170-217.5) N=4** | **0.94** |
| **Door to Groin**  **puncture†** | **55(48-60)**  **N=6** | **57.5(50-92.5)**  **N=4** | **0.27** |

| **Median (IQR)** |  |  |  |
| --- | --- | --- | --- |
| **Groin puncture to** | **15(15-15)** | **20(17.5-20)** | **0.94** |
| **recanalization†** | **N=6** | **N=4** |  |
| **Median (IQR)** |  |  |  |

# * Modified thrombolysis in cerebral infarction (TICI) grading system. †Duration in minutes.

**Table S 3: Comparison of in hospital complications**

| **Total (N=40)** | **TPA**  **(N=21)** | **TPA+NAC**  **(N=19)** | **P value** |
| --- | --- | --- | --- |
| **Days of Hospital stay**  **Mean ± SD** | **11.15±11.00** | **7.7±6.14** | **0.30** |
| **Infection during** |  |  |  |
| **hospital stay** |  |  |  |
| **Total** | **3(14.2%)** | **4(21.05%)** |  |
| **BSI** | **1(33.33%)** | **1(25%)** | **0.64** |
| **VAP** | **2(66.66%)** | **2(50%)** |  |
| **HAP** | **0** | **1(25%)** |  |
| **Ventilatory**  **requirement** | **3(14.29%)** | **2(10.53%)** | **0.90** |
| **Tracheostomy done** | **1(4.76%)** | **2(10.53%)** | **0.92** |
| **In hospital mortality** | **2(9.52%)** | **1(5.26%)** | **0.92** |

# BSI Blood stream infection; HAP Hospital acquired pneumonia; VAP Ventilator associated pneumonia

**Figure S 1. Comparison of Onset to Door time**


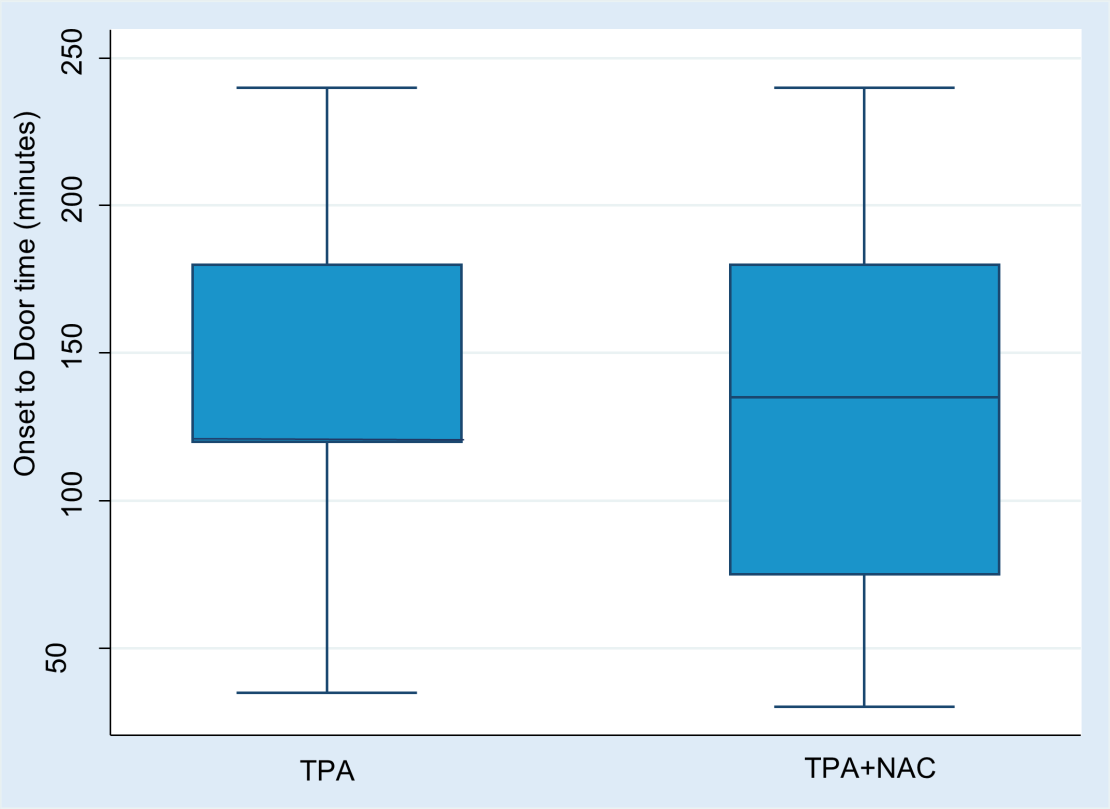


# Figure S 2: Comparison of Door to CT time


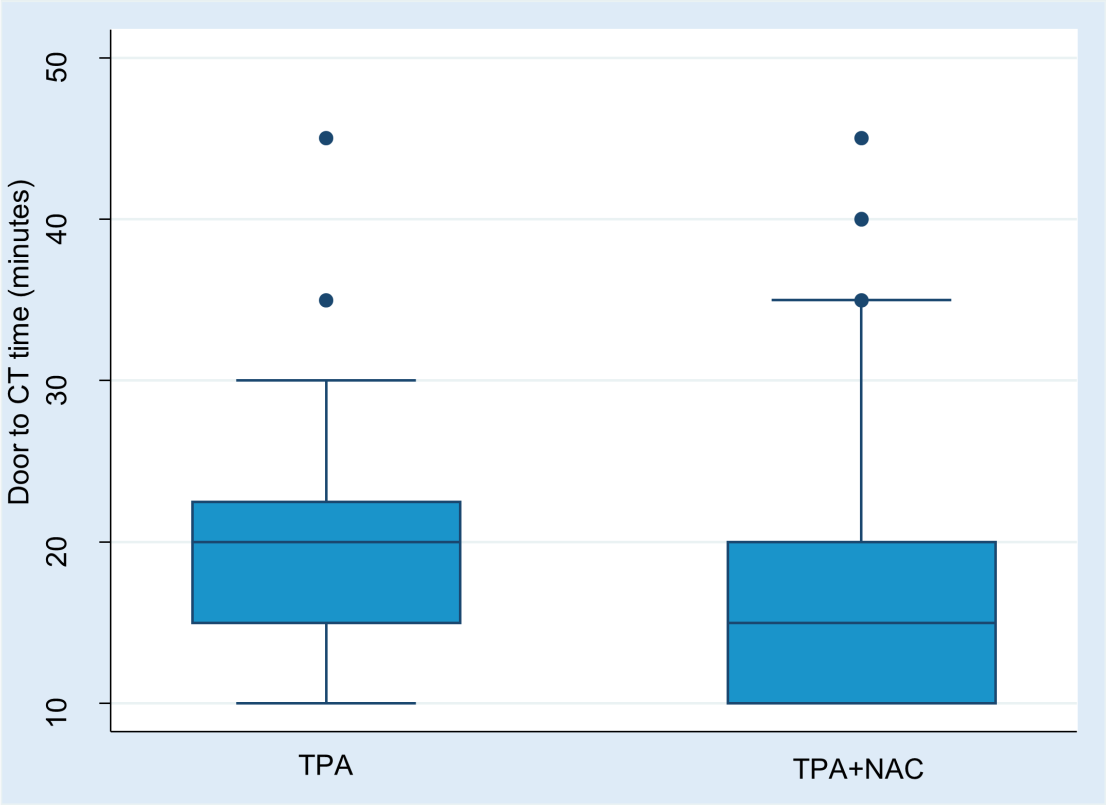


**Figure S 3: Comparison of Onset to TPA time**


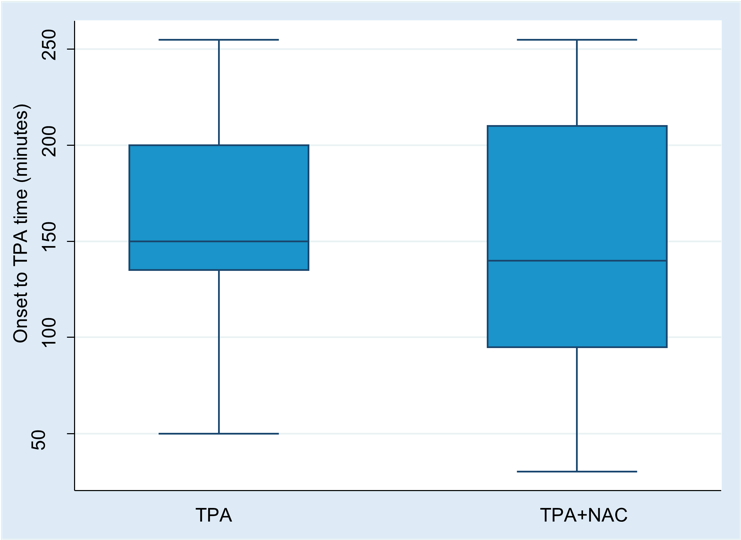


# Figure S 4: Comparison of Door to TPA time


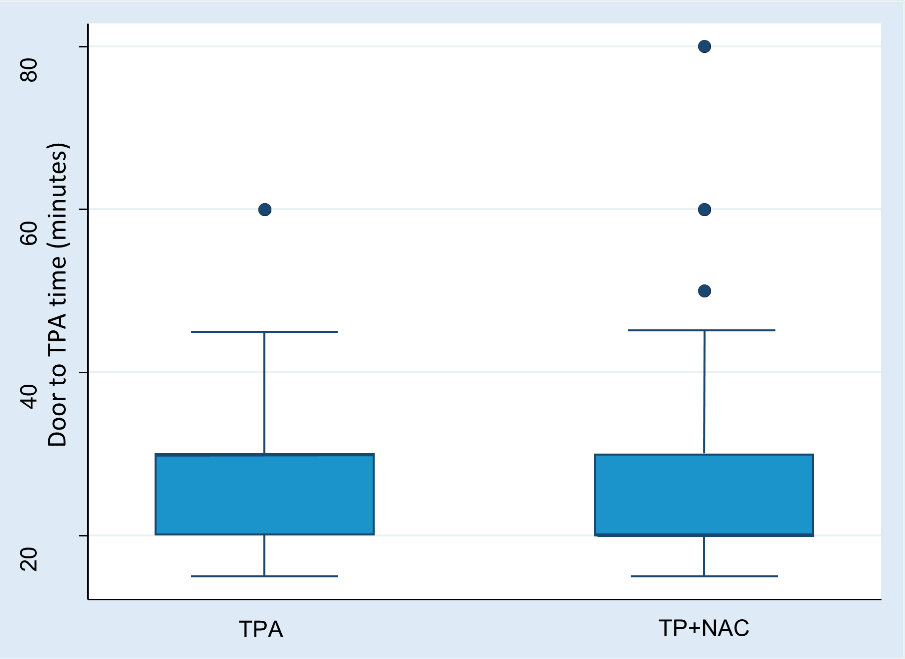


**Figure S 5: Comparison of onset to groin puncture time in patients who underwent thrombectomy.**


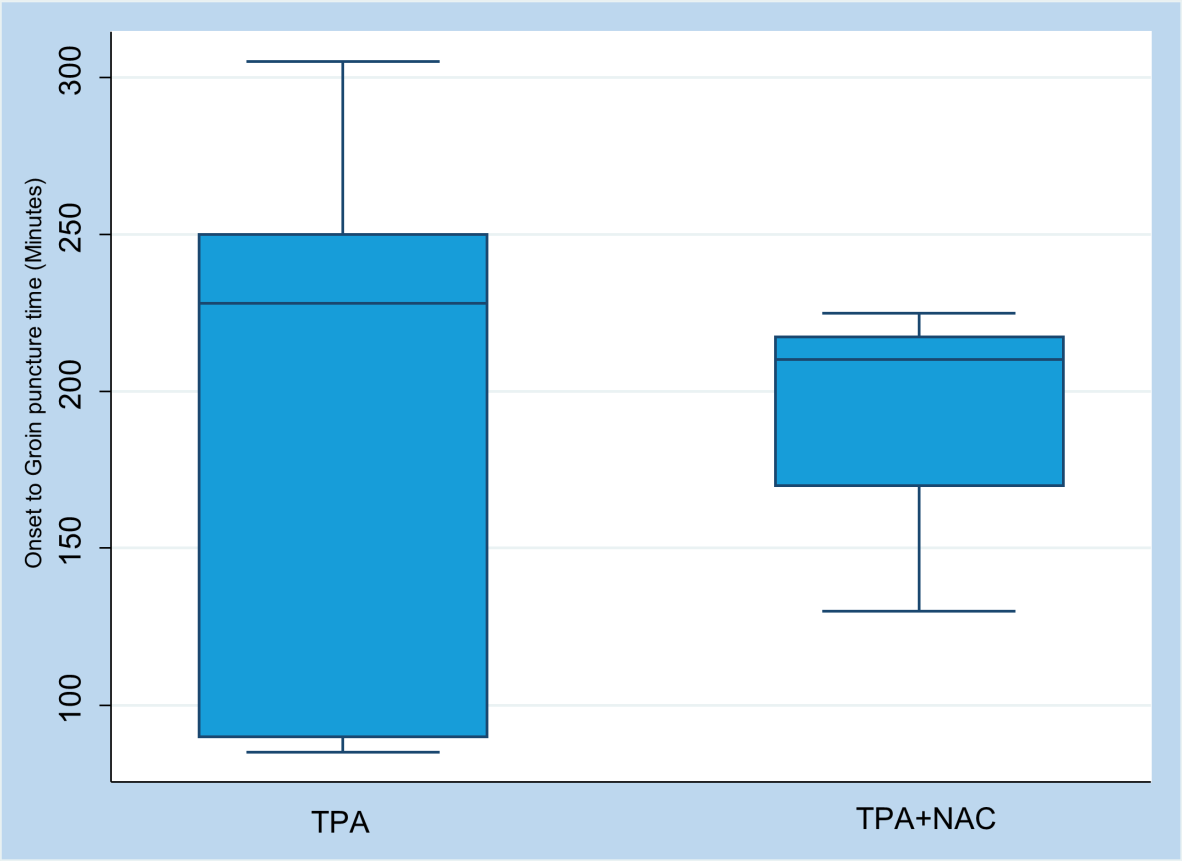


# Figure S 6: Comparison of door to groin puncture time in patients who underwent thrombectomy.


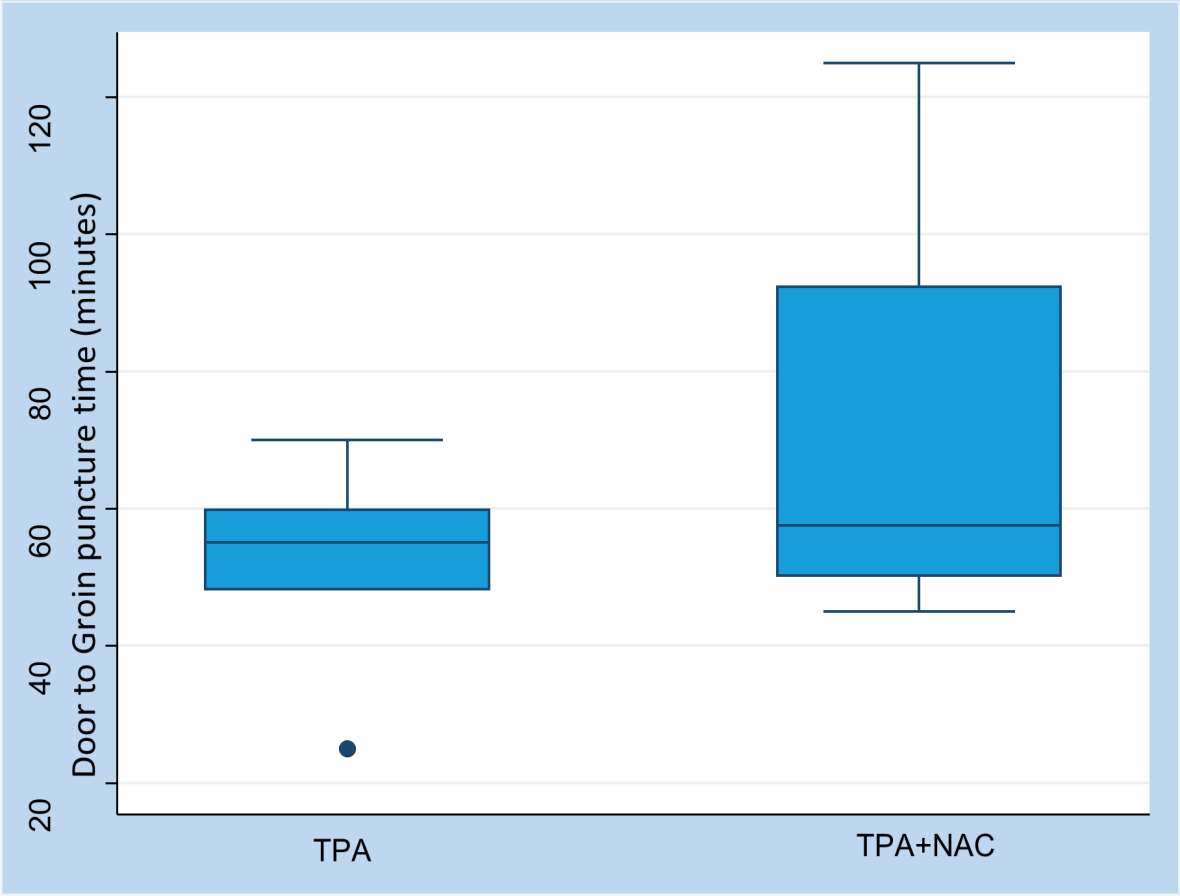

Supplement: Supplementary file 1 — Supplementary Information. [file 41598_2023_49054_MOESM1_ESM.docx]
